# Experimental study of the tissue reaction caused by the presence of cellulose produced

**DOI:** 10.1016/S1808-8694(15)30779-5

**Published:** 2015-10-19

**Authors:** Wander Lopes Amorim, Henrique Olival Costa, Flávia Coelho de Souza, Marilia Germanos de Castro, Leonardo da Silva

**Affiliations:** 1 Master's degree student, Santa casa de Sao Paulo. Otorhinolaryngologist; 2 Otorhinolaryngologist, headamp;neck surgeon. Doctor in Otorhinolaryngology, adjunct professor of the Otorhinolaryngology Department, Santa Casa de Sao Paulo, coordinator of the graduate program in Otorhinolaryngology, Santa Casa de Sao Paulo; 3 Master's degree in veterinary science, doctoral degree student, Santa Casa de Sao Paulo; 4 Professor (instructor), Santa Casa de Sao Paulo, pathologist; 5 Doctor in Otorhinolaryngology, professor (instructor), Santa Casa de Sao Paulo. Faculdade de Ciencias Médicas da Santa Casa de Sao Paulo

**Keywords:** biocompatibility, cellulose, rabbits, nose

## Abstract

Several materials have been proposed for nasal reconstruction. There is no consensus on which is the best. The cellulose blanket produced by bacteria may be a possible cartilaginous addition element to the nose.

**Aim:**

to study tissue reaction to cellulose in the dorsal nose of rabbits.

**Materials and Methods:**

22 New Zealand rabbits were used. In 20 a cellulose blanket was implanted in the nasal dorsum and 2 served as controls. They were followed up through a period of three and six months, after which their nostrils and nasal dorsums were removed and histological studies were carried out on them, considering defined parameters of inflammation such as vascular congestion, intensity of the inflammatory process and presence of purulent exudate.

**Results:**

The inflammatory process remained stable, showing its relationship with the surgical procedure and not with the presence of the cellulose blanket. There were no statistical differences in the other parameters.

**Conclusion:**

The cellulose blanket produced by *Acetobacter xylinum* presented good biocompatibility, remained stable during the entire study period, and could be considered a good material for elevating the nasal dorsum.

## INTRODUCTION

Humankind has always been interested in searching for an esthetic ideal and improving bodily contours. The nose is in a strategic central position on the face, and is thus more susceptible to trauma, which may result in facial deformity and lead to social stigma and prejudice of many sorts.

Although its main function is in breathing, the nose is also important esthetically; its central anatomical position reveals the genetic burden in facial contours.

Reconstruction of the nose has been a concern in medicine since antiquity. In the Roman Empire, prisoners of war had their noses amputated as punishment. In Ancient India, adultery and theft were punished by nasal amputation. Thus, the first reports of nasal reconstruction of deformed or mutilated noses date from these times.^1^

Modern rhinoplasty started in 1860, when the scientific community began to think about the structure of the nose. Success in the use of the maxillary and frontal bones to recompose the nasal structure led researchers to try using the ulna, tibia and ribs.^2^

Based on the first efforts in rhinoplasty, nasal reconstruction techniques have developed continuously, and currently provide excellent results. There are many options for nasal reconstruction, due to the development of stable, non-reactive and easily available alloplastic materials, among other factors.^3^

Preferred materials for supporting the dorsum of the nose should provide adequate resistance, volume and shape persistence, easy insertion and coating, and sufficient availability and ability to mimic the natural contour of the nasal dorsum.^1^^,^^2^^,^^4^, ^5^, ^6^, ^7^

Many materials have been proposed for nasal reconstruction. There is, however, no consensus about which is superior. Further studies are needed to seek new materials or substances that have never been used in this application, or that have been used in other parts of the human body, and that might become feasible solutions for reasons of practicality, economy and decreased comorbidity.

**Figure 1:**
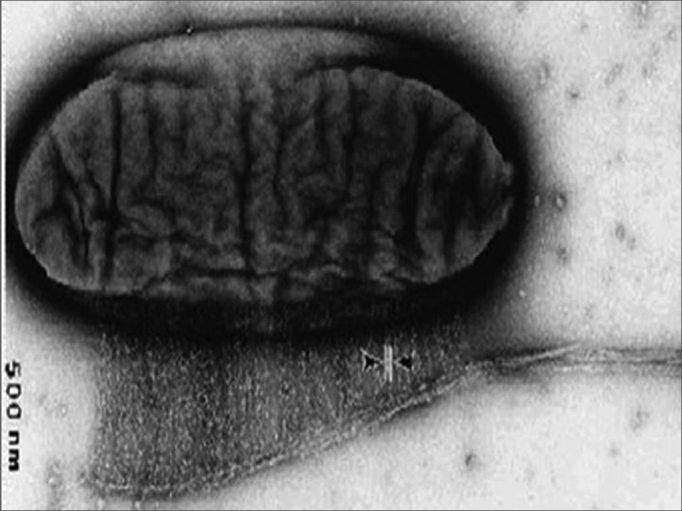
Microscopy of Acetobacter producing cellulose

In 1984, Luis Fernando Xavier Farah, a microbiologist, developed an economically feasible industrial process for producing cellulose based on the fermentation of bacteria of the Acetobacter genus. After processing, the resulting membrane has selective permeability; it is permeable to water vapor but not to microorganisms. It is homogeneous, its mean thickness is 0.05 mm, and it contains no adhesives or additives. It consists basically of cellulose, which is inert, resistant and insoluble in all organic solvents. Additional specific physical features include: defined permeability to liquids and gases, tensile and traction resistance, and characteristic and stable molecular weight and structure. Cellulose membranes have been tested in a variety of applications, from artificial skin to bullet-proof vests, computer screens and paper for preserving historical documents.^8^ (Fig. 1)

In searching for alternatives to reconstruct the dorsum of the nose, we decided to study healing when applying a bacterial (Acetobacter xylinum) cellulose sponge produced by Bionext® for raising the nasal dorsum. We wished to increase the volume of the dorsum without changing the usual texture and esthetic consistency and to avoid the disadvantages or harvesting and tolerability of a graft material.

The Bionext® cellulose sponge (ANVISA N 80255120001) is produced by Bionext Produtos Biologicos. It consists of a flexible, semitransparent, yellowish membrane composed of polysaccharides synthesized by bacteria of the genus Acetobacter. It is biodegradable, non-toxic, non-pyrogenic, and can be sterilized; it has been used successfully as a temporary skin graft.^9^, ^10^, ^11^

## OBJECTIVE

The purpose of this study was to assess the tissue response in rabbits to the presence of cellulose produced from bacteria (Acetobacter xylinun) as a material for elevating the dorsum of the nose.

## MATERIAL AND METHOD

The Research Ethics Committee of the Instituto de Ciências Avançadas em Otorrinolaringologia approved this study on 13 June 2006. The study was undertaken in a vivarium at this institute, and was supervised and monitored by a veterinarian. The animals were placed in appropriate cages and had free access to water and a standardized commercial ration.

Surgical procedures were done according to the Ethics Committee guidelines for the Experimental Surgery Unit (Unidade de Técnica Cirúrgica Experimental) of the Santa Casa de Sao Paulo, the norms in the Federal Law number 6 638 (8 May 1979) and the ethical principles for experiments of the Brazilian Code for Animal Experiments (Codigo Brasileiro de Experimentacao em Animais or COBEA).

### Sample Size and Selection

Twenty-two male New Zealand rabbits aged 6 months were studied during three to six months. Study groups were established randomly according to the follow-up time. The choice of this animal was based on the facts that they are easy to handle, monitor and assess.

Animal groups were chosen as follows:

a- 2 rabbits for control purposes of the surgical procedure;
b- 20 rabbits for the study group; 10 were assessed 3 months after surgery, and another 10 were assessed 6 months after surgery;
c- the choice of animals for the follow-up groups was made by a random draw on the day of euthanasia.

### Preparation of the Material

**Figure 2:**
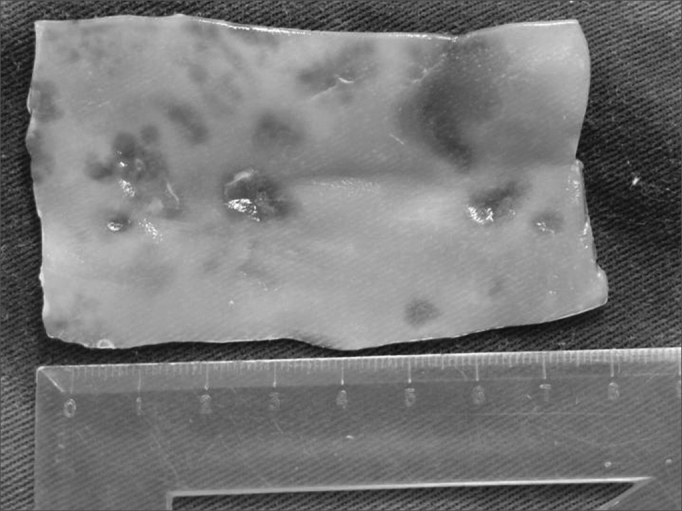
Cellulose membrane before folding for insertion

Before surgery, the cellulose sponge was sterilized in a glutaraldehyde solution for 10 minutes and washed in saline. A strip measuring 4 cm long and 1 cm wide was cut, prepared, folded and molded to become an elevation element to be inserted in the dorsum of the nose (Fig. 2).

### Surgical Procedure

General anesthesia was done following a 4-hour fasting period. All animals were anesthetizes with ketamine 40 mg/kg and xylazine hydrochloride 10 mg/kg intraperitoneally, and were ventilated mechanically throughout the procedure.

The fur was removed from the skin over the dorsum of the nose to standardize the conditions for photography, to note volume expansion and to evaluate the clinical status of the cellulose graft.

A 1 cm incision was made horizontally on the frontal area and dissection was done subperiotally from the interorbitary incision towards the tip of the nose to attain a 7 cm × 1 cm insertion tunnel. The same procedure was done in the controls, without inserting the cellulose sponge, to note the response to the procedure.

### Placing the Cellulose in the Dorsum of the Nose

A strip measuring 4 cm length by 1 cm width, which had been previously designed and cut, was placed in the tunnel made over the dorsum of the nose.

After insertion of the cellulose sponge in the tunnel, 3.0 mononylon sutures were done to close the frontal incision; at this point the procedure was ended. The animals were given 1 ml intramuscular benzilpenicillin benzatin and 0.2 ml dipyrone.

**Figure 3:**
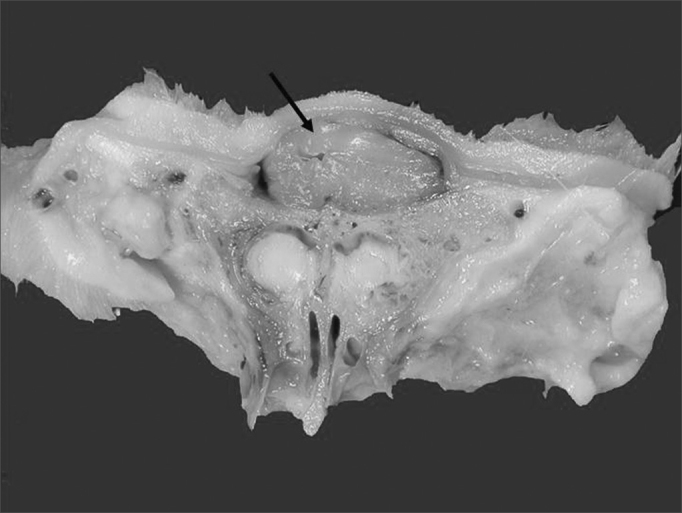
Sagittal section of specimen showing cellulose included in the dorsum of the nose (arrow)

### Euthanasia

After a 3-month follow-up period, 10 rabbits were chosen for euthanasia by a draw among those that received the graft and 1 rabbit from the control group. Animals were given an intracardiac injection of potassium chloride while under anesthesia. An incision was made along the lateral rima oris, around the rostral margin of the orbit until reaching the frontal bone; a specimen of the rostral area of the animal was removed. The dorsum and lateral portion of the nose from the nostrils to the frontal area were kept intact.

Specimens were stored in a 10% formaldehyde solution for anatomy and pathology studies. A similar procedure was done after a 6-month follow-up period.

### Histopathology

The anatomical specimens were frozen and decalcified, after which serial coronal sections were made at 5 mm from the tip of the nose to the frontal suture. Inflammation, the graft thickness and the graft-host relation were studied.

An otorhinolaryngologist and a pathologist standardized the parameters that were to be observed. One pathologist analyzed the slides without knowing to which group each slide belonged. The sections were hematoxyllin-eosin (HE) stained for histology.

An optical microscope was used. The following parameters for defining inflammation were evaluated and graded:

### Vascular congestion

0- mild
1- moderate
2- intense

### Intensity of inflammation

0- absent
1- mild
2- moderate
3- intense

### Pus

0- absent
1- present

### Status of the cellulose sponge

0- absent
1- intact
2- partially fragmented
3- fragmented

**Figure 4:**
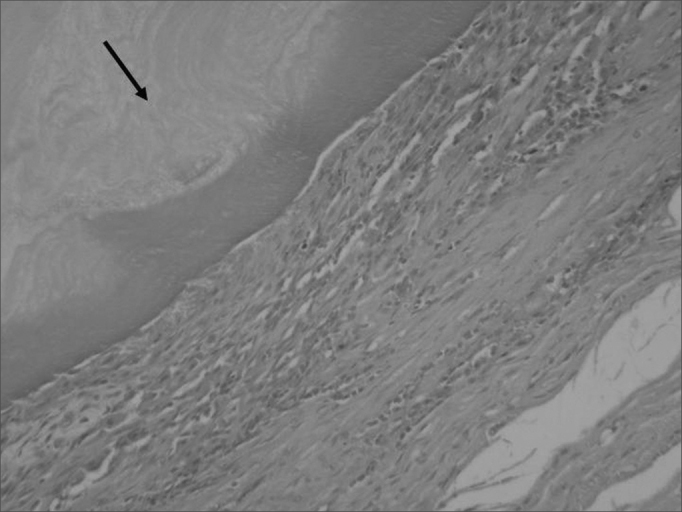
Histology showing cellulose (arrow) in contact with subcutaneous tissue. HE, 40X

Digital photography was used to record images of all slides (Fig. 4).

### Macroscopic Evaluation

A macroscopic parameter was used in assessing the elevation of the dorsum of the nose after placing the cellulose sponge; this parameter consisted of observing the profile of the animal 3 and 6 months after surgery.

Elevation of the dorsum:

1- Flat dorsum
2- Elevated dorsum

### Statistical Analysis

Results of the anatomical and pathologic examinations were tabulated as categorical variables and compared using the Mann-Whitney non-parametric test (independent samples) to assess the intervention pairs.

The Mann-Whitney test is a non-parametric test similar to the T-test for independent samples. It is used when the distribution of results is not normal and cannot be distributed using a logarithmic transformation. The test combines and classifies the results of two samples and calculates the statistical difference among the sum of rankings. A 5% value was considered statistically significant (P<0.05).

## RESULTS

Tables 1 and 2 show the histological findings of inflammation, as represented by the preestablished parameters (vascular congestion, pus, acute inflammation and status of the cellulose sponge), and the macroscopic findings of the elevated nasal dorsa.

**Table 1:** Macroscopic and microscopic parameters assessing inflammation and the status of the cellulose sponge after 3 months follow-up.

Rabbit	External aspect of dorsum	Status of cellulose	VC	P	AI
1	Elevated	Intact	0	0	1
2	Elevated	Intact	1	0	1
3	Elevated with partially necrotic skin	Partially fragmented	0	1	3
4	Elevated	Intact	2	0	2
5	Elevated	Partially fragmented	1	0	2
6	Elevated	Intact	0	0	1
7	Elevated	Partially fragmented	1	0	1
8	Elevated with partially necrotic skin	Partially fragmented	2	2	3
9	Elevated	Intact	0	0	1
10	Elevated	Partially fragmented	2	0	3
11*	–	–	–	–	–
12**	–	–	–	–	–

VC–vascular congestion; 0: mild; 1: moderate; 2: intense.

P–pus 0: absent; 1: present.

AI–inflammation 0: absent; 1: mild; 2: moderate; 3: intense.

* this animal died in laboratory a few days after the procedure.

** this animal was sacrificed, the specimen was sent to pathology but was lost.

**Table 2:** Microscopic and macroscopic parameters for assessing inflammation and the status of the implanted cellulose sponge after 6 months.

Rabbit	External aspect of dorsum	Status of cellulose	VC	P	AI
1	Elevated	Partially fragmented	2	1	2
2	Elevated	Partially fragmented	1	0	1
3	Elevated	Partially fragmented	0	0	2
4	Elevated	Partially fragmented	0	0	1
5	Elevated	Partially fragmented	0	0	2
6	Elevated	Partially fragmented	0	0	2
7	Elevated with partially necrotic skin	Partially fragmented	0	0	1
8	Elevated	Partially fragmented	0	0	2
9	Elevated	Partially fragmented	0	0	0
10	Elevated	Partially fragmented	0	0	0
11*	–	–	–	–	–

VC–vascular congestion 0:mild, 1:moderate, 2: intense

P–pus 0: absent, 1: present

AI–inflammation 0: absent, 1: mild, 2: moderate, 3: intense

*** animal died in laboratory a few days after the procedure.

The macroscopic assessment of the elevated dorsa showed that all animals had an elevated dorsum by the end of three months; the skin was partially necrotic in two of 10 animals. Similarly, all animals had an elevated dorsum by the end of six months; in this case, the skin was partially necrotic in one animal.

Based on the preestablished parameters, microscopy revealed that the cellulose sponge was intact in five animals by the end of three months; the material was partially fragmented in another five animals. There was partial fragmentation of the cellulose sponge by the end of six months, but this finding was not statistically significant (p = 0.065).

The assessment of inflammation showed that there was no statistically significant difference in vascular congestion between the 3-month group and the 6-month group (p = 0.279); likewise, there was no statistically significant difference in the presence of pus between the 3-month and 6-month group (p = 0.684).

*The Mann-Whitney test* for independent samples

### Vascular Congestion

**Table:** 

Mean rank of the 90-day group	12,00
Mean rank of the 180-day group	9,00
p-value	0,279

The test results indicated that there was no significant difference (p-value > 0.05) in vascular congestion at 90 and 180 days.

The parameter inflammation did not differ statistically between the 3-month and the 6-month group (p = 0.317).

### Pus

**Table:** 

Mean rank of the 90-day group	11,05
Mean rank of the 180-day group	9,95
Valor-p	0,684

The test results indicated that there was no significant difference (p-value > 0.05) in pus at 90 and 180 days.

### Inflammation

**Table:** 

Mean rank of the 90-day group	11,75
Mean rank of the 180-day group	9,25
Valor-p	0,317

The test results indicated that there was no significant difference (p-value > 0.05) in inflammation at 90 and 180 days.

### Elevation of the dorsum

**Table:** 

Mean rank of the 90-day group	10,50
Mean rank of the 180-day group	10,50
Valor-p	1

The test results indicated that there was no significant difference (p-value = 1) in elevation of the nasal dorsum at 90 and 180 days.

### Status of cellulose

**Table:** 

Mean rank of the 90-day group	7,75
Mean rank of the 180-day group	12,50
Valor-p	0,065

The test results indicated that there was no significant difference (p-value > 0.05) in cellulose status at 90 and 180 days.

## DISCUSSION

Otorhinolaryngological studies on the feasibility of using bacterial cellulose in mucosal tissues, such as the nasal septum, the turbinates and as substitutes for tympanic membranes have been undertaken and appear promising. Material with such properties has not been duly investigated in patients undergoing nasal remodeling. As animal tests have not included an evaluation of this material in the region of interest, we found that it was necessary to ascertain the healing conditions and efficacy of cellulose sponges as a material for elevating the nasal dorsum of rabbits.

The surgical technique for implanting cellulose in the nasal dorsum of rabbits was feasible and easily done. A frontal interorbitary incision with subperiosteal detachment to the tip of the nose made it possible to construct a tunnel for easily placing the implant material. Increased sensitivity was noted on the tip of the nose in some animals, which did not impede constructing the tunnel for inserting a cellulose sponge.

The main concerns in this study were to establish whether the material was biocompatible–if the inflammatory response would be tolerable–and to assess if the elevated dorsum would remain unaltered with time.

In the literature we found a variety of methods for assessing graft biocompatibility in a host. Such methods are: tissue and cell culture, histochemical analysis,^12^ biochemical studies,^13^^,^^14^ histological studies, and perfusion studies of a whole organ. There are also measures of weight, stiffness, elasticity, elongation, mechanical breakages, and surface changes revealed by electron microscopy. More recently, radiological exams–such as computed tomography and magnetic resonance imaging with or without specific radioactive markers–have been used in assessing tissue responses to metallic implants that may dissolve and cause inflammation.^14^, ^15^, ^16^, ^17^ However, the most common method used in experimental studies is histological analysis of hematoxilin-eosin stained specimens.^18^, ^19^, ^20^, ^21^, ^22^, ^23^, ^24^, ^25^, ^26^, ^27^, ^28^

We chose the histological analysis because it is a simpler method and provides general information about tissue responses to implanted materials.

The fact that cellulose did not exacerbate or prolong the inflammatory process may be explained by its biocompatibility, which results in a more favorable tissue reaction that does not perpetuate inflammation, as shown in various published reports.^13^^,^^14^^,^^29^^,^^30^

Biocompatibility was investigated by observing local inflammation. Vascular congestion and pus were rarely present.

The inflammation encountered in the third month of follow-up did not change significantly by the sixth month; it was thus related with the surgery rather than the presence of the cellulose sponge. Inflammation involves participation of cells in tissue repair. There were no signs of an increased inflammatory response in operated animals with grafts compared to those in which cellulose was not placed. In animals with the cellulose graft, a polymorphonuclear inflammation with the presence of multinucleated giant cells was observed. There was not chronic inflammation with macrophages and lymphocytes, neither granuloma formation after the appearance of giant cells, which would be typical of an immune response and macrophage sequestration.

An assessment of the final quality of the graft as a material for elevating the nasal dorsum showed that there was a marked immediate postoperative change in the structure of the nasal dorsum that persisted until the end of follow-up. All animals in both groups had an elevated dorsum (p=1).

Histology of the graft revealed that there was a tendency for fragmentation to occur with time; there was, however, no statistical difference between the three-month and six-month groups (p=0.065). Fragmentation was not accompanied by macrophagia; thus, there was no cellulose resorption within the follow-up period. Loss of properties of the implanted material could suggest lack of biocompatibility; the tissue response, however, did not appear unsatisfactory to be considered as such. Cellulose fragmentation may also be seen as a positive sign that the material is being incorporated into tissues with no excessive or pathological inflammation.

Material biocompatibility studies generally require assessments in different periods, given the pathophysiology of tissues responses to foreign bodies. The progression time was important for an evaluation to be made of the cellulose sponge, local inflammation, and the status of the nasal dorsum within the follow-up period. As there are no other published papers in the literature about the use of this material for the abovementioned end, there is no possibility at the moment for a comparative analysis. Possibly further research with longer follow-up times may provide additional information about fragmentation–which was seen in some samples–and whether the elevation of the nasal dorsum might be affected eventually.

In this study we were able to see that the cellulose sponge was adequately malleable and easily handled. Once inserted in a subcutaneous pouch, it provided a natural consistency to the nasal dorsum and an excellent elevation of the nasal profile. Its physical features and biocompatibility, and the fact that this material is easily placed and may probably be modeled during insertion, make of this product a possible candidate for treatments that required an addition of cartilage and/or bone.

## CONCLUSION

The cellulose sponge made by Acetobacter xylinum was biocompatible and remained stable as the study progressed. It may be considered as a good material for use in nasal dorsum elevation in rabbits.
